# Empiric antibiotic prescribing practices for gram-positive coverage of late-onset sepsis in neonatal intensive care units in North America

**DOI:** 10.1017/ice.2024.176

**Published:** 2025-01

**Authors:** Dara Simcha Petel, Sandra Isabel, Kyong-Soon Lee, Joseph Yuk Ting, David A Kaufman, Pablo Jose Sanchez, Sarah Khan, Kathryn Timberlake, James Wright, Michelle Science

**Affiliations:** 1 Division of Infectious Diseases, Department of Paediatrics, The Hospital for Sick Children, Toronto, ON, Canada; 2 Axe Maladies infectieuses et immunitaires, Centre de recherche du CHU de Québec-Université Laval, Québec, QC, Canada; 3 Division of Neonatology, Department of Paediatrics, The Hospital for Sick Children, Toronto, ON, Canada; 4 Division of Neonatology, Department of Pediatrics, University of Alberta, Edmonton, AB, Canada; 5 Division of Neonatology, Department of Pediatrics, University of Virginia School of Medicine, Charlottesville, VA, USA; 6 Department of Pediatrics, Divisions of Neonatology and Pediatric Infectious Diseases, Nationwide Children’s Hospital, The Abigail Wexner Research Institute at Nationwide Children’s Hospital, Center for Perinatal Research, Ohio Perinatal Research Network, The Ohio State University College of Medicine, Columbus, OH, USA; 7 Division of Infectious Diseases, Department of Paediatrics, McMaster University, Hamilton, ON, Canada; 8 Department of Pharmacy, The Hospital for Sick Children, Toronto, ON, Canada; 9 Public Health Ontario, Toronto, ON, Canada

## Abstract

Late-onset sepsis (LOS) in the neonatal intensive care unit (NICU) causes significant morbidity and mortality, yet guidance on empiric management is limited. We surveyed NICUs across Canada and the United States regarding their empiric antimicrobial regimens for LOS, thereby identifying large practice variations and high rates of empiric vancomycin use.

## Background

Late-onset sepsis (LOS) in the neonatal intensive care unit (NICU) is associated with significant morbidity and mortality.^1^ The most common etiology remains coagulase-negative staphylococci (CoNS), recently accounting for 43% of central line-associated bloodstream infections in Canadian NICUs.^2^

There is a paucity of data guiding empiric antibiotic regimens for LOS. Given provider concerns regarding CoNS resistance to beta-lactam antibiotics and methicillin-resistant *Staphylococcus aureus* (MRSA), vancomycin is often prescribed empirically to provide gram-positive coverage.^3,4^ The frequent use of vancomycin raises a need for alternative regimens such as anti-staphylococcal penicillin agents (eg, cloxacillin, oxacillin, nafcillin) or first-generation cephalosporin agents (eg, cefazolin). A recent retrospective review pre- and post-implementation of a vancomycin reduction guideline in the United States found nafcillin to be a safe alternative to vancomycin for initial empiric therapy of LOS in patients without a history of MRSA infection or colonization.^5^

This study aimed to understand empiric antibiotic prescribing practices for gram-positive coverage of LOS in Canadian and American NICUs, thereby informing opportunities for antibiotic stewardship efforts.

## Methods

An electronic survey was distributed in July 2023 to 71 NICUs, 33 (46%) in Canada and 38 (54%) in the United States. We requested responses reflective of institutional rather than individual practices. Duplicate responses were reconciled. Survey questions focused on empiric antibiotic prescription practices for LOS, defined in this study as sepsis occurring after 72 hours of age among infants in the NICU.^5^ Adjustments to empiric regimens based on critical illness and/or the presence of a central venous catheter (CVC) were assessed. Critical illness was not further defined; the threshold for meeting this definition was left to the discretion of the respondent. Demographic information, MRSA screening practices, MRSA prevalence, and CoNS resistance rates to anti-staphylococcal penicillin agents were also obtained. Where MRSA and/or CoNS resistance rates were unknown, email requests for this information were sent to the hospital’s infection prevention and control team.

Descriptive statistics were used to summarize survey results. Chi-square tests assessed for differences in empiric vancomycin use by NICU size, MRSA positivity rate, anti-staphylococcal resistance rate and hospital location. A *P*-value <0.05 was considered statistically significant. The study was approved as a quality improvement initiative by The Hospital for Sick Children Quality Committee.

## Results

Survey responses were received from 53.5% (38/71) of sites; 48.5% (16/33) Canadian; and 57.9% (22/38) American (Supplementary Table 1). Most respondents were neonatologists (89.5%) from level 4 (regional) NICUs with on-site surgical capability (76.3%).

Most centers either screen all or selected patients for MRSA on admission (36.8%, 39.5% respectively) (Supplementary Table 1). The majority of centers (52.6%) reported MRSA positivity rates of less than 5%. CoNS anti-staphylococcal penicillin resistance rates of >50% were reported by 34.2% of centers, while 23.7% reported rates of <5%.

Nine centers (23.7%) reported empiric vancomycin use in all LOS cases; others reported empiric vancomycin use in cases of known MRSA colonization (57.9%), critically-unwell patients (42.1%), previous CoNS sepsis (21.1%), current CVC (21.1%), CVC placements within 48 hours (18.4%), or multiple recent vascular access attempts (2.6%). Empiric vancomycin use was not associated with NICU size, MRSA positivity rate, CoNS anti-staphylococcal penicillin resistance rate, or hospital location; a higher proportion of empiric vancomycin was reported among American centers (7/22; 31.8%) and level 4 (regional) NICUs (8/29; 27.6%), though this was not statistically significant (Table 1).

**Table 1.** Empiric vancomycin use for late-onset sepsis

Variable	N (%)	*P*-value
**Level NICU**		0.58
Level 4 (Regional NICU)	8/29 (27.6)	
Level 3 (NICU)	1/8 (12.5)	
Level 2 (Special care nursery)	0/1 (0.0)	
**NICU MRSA positivity rate**		0.39
<5%	4/20 (20.0)	
5%–15%	0/4 (0.0)	
Screening not performed	3/9 (33.3)	
**NICU CoNS anti-staphylococcal penicillin resistance rate**		0.09
<5%	1/9 (11.1)	
5%–15%	0/1 (0.0)	
15%–30%	1/1 (100.0)	
30%–50%	0/3 (0.0)	
>50%	1/13 (7.7)	
Testing not performed	1/2 (50.0)	
**Hospital location**		0.17
Canada	2/16 (12.5)	
USA	7/22 (31.8)	

NICU, neonatal intensive care unit; MRSA, methicillin-resistant *Staphylococcus aureus*; CoNS, coagulase-negative staphylococci.

**Figure 1.**
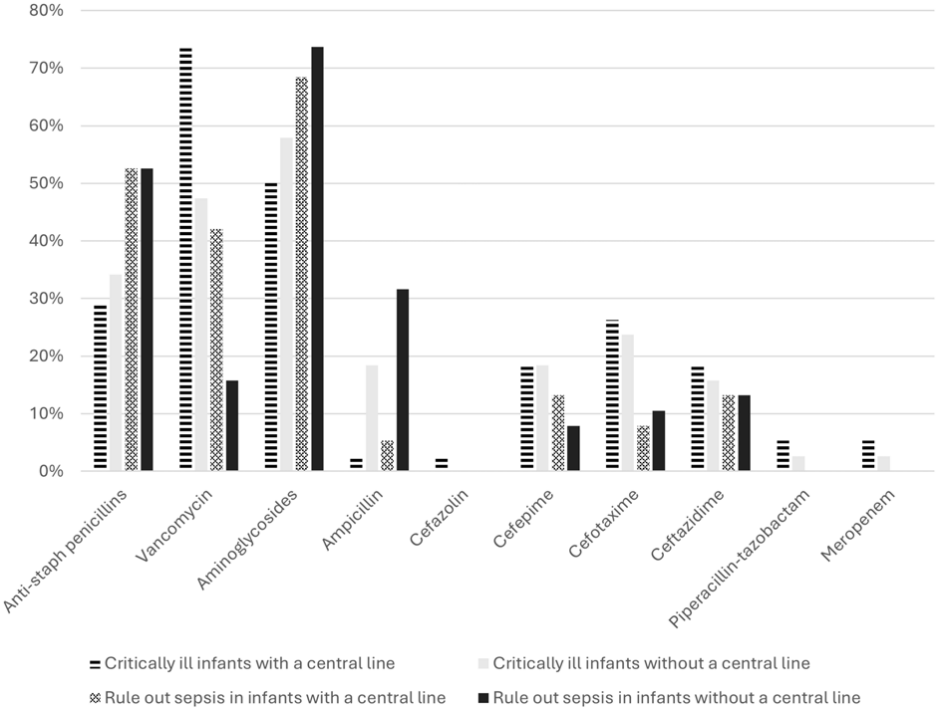
Empiric antibiotic prescribing practices for suspected late-onset sepsis. Legend: Empiric antibiotic prescribing rates for late-onset sepsis depending on the presence or absence of a central venous catheter (CVC) and the clinical status of the patient. The highest rate of vancomycin initiation was reported among critically-ill infants with a CVC, at 76%.

In critically-unwell patients with a CVC, 73.7% and 28.9% of sites reported empiric vancomycin and anti-staphylococcal penicillin use for gram-positive coverage, respectively (Figure 1). This contrasts with responses for critically-unwell patients without a CVC, where 47.4% of sites reported empiric vancomycin and 34.2% reported empiric anti-staphylococcal penicillin use. For rule-out sepsis patients, these responses changed to 42.1% empiric vancomycin use and 52.6% empiric anti-staphylococcal penicillin use for those with a CVC, compared to 15.8% empiric vancomycin and 52.6% empiric anti-staphylococcal penicillin use for those without a CVC. Of note, substantial use of empiric third and fourth generation cephalosporins was reported, in up to 63.2% of critically-ill neonates and 34.2% of those undergoing rule-out sepsis evaluations.

## Discussion

The significant variation in antimicrobial regimens for LOS highlights the lack of standardization of LOS management across Canadian and American NICUs. Initiation of vancomycin or an anti-staphylococcal penicillin for empiric gram-positive coverage was both center- and case-dependent.

The safety of anti-staphylococcal penicillins rather than vancomycin to provide empiric gram-positive coverage during LOS evaluation in centers with low MRSA prevalence has been demonstrated internationally, without evidence of increased duration of bacteremia, infectious complications or mortality.^5–7^ Despite this evidence, institutional practices have not consistently changed to reduce unnecessary empiric vancomycin use. This presents a major target for antimicrobial stewardship interventions given the high frequency of suspected LOS and its significant contribution to empiric vancomycin utilization. Vancomycin use has major pitfalls; multiple doses may be required to achieve therapeutic levels, frequent drug level monitoring is indicated, and risks include acute kidney injury and the development of resistant organisms. Vancomycin may also be less effective than beta-lactams for treatment of MSSA, an important consideration given that MSSA is more common than MRSA infections in NICUs.^3,8^ There is precedent for level 4 (regional) NICUs successfully reducing vancomycin prescribing, in one case utilizing a multimodal approach of education, clinical pathway development, 48-hour antibiotic time-outs and prospective audit with feedback derived using a quality improvement framework.^9^

The reasons for low uptake of anti-staphylococcal penicillin agents across NICUs remain unclear, but contributory factors may include lack of a neonatal society guidance document and hesitancy to change practice. Other factors include a perceived need to empirically cover for CoNS, a major cause of LOS which tends to be resistant to anti-staphylococcal penicillin agents, concern for MRSA and *Enterococcus* spp., and risk of chemical phlebitis with cloxacillin intravenous administration. Cefazolin has similar constraints, limited blood-brain-barrier penetration and less familiarity as empiric LOS therapy.

Despite concerns for CoNS in critically-unwell patients, lack of empiric CoNS coverage has not been associated with adverse outcomes, as CoNS have not been associated with fulminant sepsis, typically grow within 24–48 hours allowing for rapid adjustment in therapy if indicated, and may reflect blood culture contamination.^3,4^ A retrospective review of infants with CoNS bloodstream infection admitted to 348 NICUs between 1997–2012 showed no mortality difference between babies treated with empiric versus delayed vancomycin therapy.^10^

The high rate of empiric third and fourth generation cephalosporin prescriptions was unexpected and warrants further evaluation. In centers where these agents are used, one could argue that additional gram-positive coverage, such as with vancomycin, may not be needed.

This survey focused largely on empiric gram-positive coverage, potentially confounding interpretation if broader spectrum antibiotics were chosen for gram-negative considerations. Additionally, MRSA prevalence was unavailable to about one-third of respondents, potentially affecting reported vancomycin use. Survey distribution was mainly to academic centers, limiting generalizability. Practice variations within a NICU would not be captured in this survey, and the small sample size limited more in-depth statistical analysis.

This survey provides insight into the wide variation in empiric antimicrobial prescribing practices for LOS in Canadian and American NICUs, thereby serving as a basis to support antimicrobial stewardship programs in streamlining the approach to this issue. Reducing vancomycin use is an important antimicrobial stewardship target; prioritization of anti-staphylococcal penicillin agents for empiric LOS therapy in the NICU should be considered.

## Supporting information

Petel et al. supplementary material 1 Petel et al. supplementary material

Petel et al. supplementary material 2 Petel et al. supplementary material

## Supplementary material

The supplementary material for this article can be found at https://doi.org/10.1017/ice.2024.176
