# Supplementary material for: Empiric antibiotic prescribing practices for gram-positive coverage of late-onset sepsis in neonatal intensive care units in North America
Source: Infect Control Hosp Epidemiol. 2024 Nov 7;46(1):102–4. doi: 10.1017/ice.2024.176 (PMC11717478; doi:10.1017/ice.2024.176)
Supplement: Petel et al. supplementary material 1 — Petel et al. supplementary material [file S0899823X24001764sup001.docx]

Supplementary Table 1. Demographics and characteristics of respondent sites

| **Demographics** | **Total (N=38), N (%)** | **Canada (N=16), N (%)** | **USA (N=22), N (%)** |
| --- | --- | --- | --- |
| **Professional role of respondents** |  |  |  |
| Neonatologist | 34 (89.5) | 13 (81.3) | 21 (95.5) |
| Neonatal nurse practitioner | 1 (2.6) | 1 (6.3) | 0 (0.0) |
| Pharmacist | 1 (2.6) | 1 (6.3) | 0 (0.0) |
| Infection prevention and control practitioner | 1 (2.6) | 0 (0.0) | 1 (4.5) |
| Quality coordinator | 1 (2.6) | 1 (6.3) | 0 (0.0) |
| **Level NICU** |  |  |  |
| Level 4 (Regional NICU) | 29 (76.3) | 7 (43.8) | 22 (100.0) |
| Level 3 (NICU) | 8 (21.1) | 8 (50.0) | 0 (0.0) |
| Level 2 (Special care nursery) | 1 (2.6) | 1 (6.3) | 0 (0.0) |
| **NICU admissions per year** |  |  |  |
| 200-400 | 6 (15.8) | 1 (6.3) | 5 (22.7) |
| 401-600 | 10 (26.3) | 4 (25) | 6 (27.3) |
| 601-800 | 7 (18.4) | 2 (12.5) | 5 (22.7) |
| 801-1000 | 6 (15.8) | 4 (25) | 2 (9.1) |
| >1000 | 8 (21.1) | 4 (25) | 4 (18.2) |
| I don’t know | 1 (2.6) | 1 (6.3) | 0 (0.0) |
| **NICU number of beds**  <20  20-40  41-60  61-80  81-100  >100 | 2 (5.3)  14 (36.8)  13 (34.2)  4 (10.5)  2 (5.3)  3 (7.9) | 1 (6.3)  10 (62.5)  3 (18.8)  2 (12.5)  0 (0.0)  0 (0.0) | 1 (4.5)  4 (18.2)  10 (45.5)  2 (9.1)  2 (9.1)  3 (13.6) |
| **MRSA screening practices** |  |  |  |
| Patients transferred to NICU from an outside hospital, emergency department, or home | 15 (39.5) | 11 (68.8) | 4 (18.2) |
| All patients on admission | 14 (36.8) | 2 (12.5) | 12 (54.5) |
| Do not screen | 7 (18.4) | 1 (6.3) | 6 (27.3) |
| Selected patients, by proximity to a case of MRSA recognized in the NICU | 6 (15.8) | 5 (31.3) | 1 (4.5) |
| All patients, when a case of MRSA is recognized in the NICU | 5 (13.2) | 5 (31.3) | 0 (0.0) |
| Screening is performed, but unsure which patients are screened | 1 (2.6) | 1 (6.3) | 0 (0.0) |
| Unsure if screening is performed | 1 (2.6) | 1 (6.3) | 0 (0.0) |
| Other | 6 (15.8) | 1 (6.3) | 5 (22.7) |
| **NICU MRSA positivity rate** |  |  |  |
| <5% | 20 (52.6) | 8 (50.0) | 12 (54.5) |
| 5-15% | 4 (10.5) | 2 (12.5) | 2 (9.1) |
| Screening not performed | 9 (23.7) | 2 (12.5) | 7 (31.8) |
| Don’t know | 5 (13.2) | 4 (25) | 1 (4.5) |
| **NICU CoNS anti-staphylococcal penicillin resistance rate** |  |  |  |
| <5% | 9 (23.7) | 2 (12.5) | 7 (31.8) |
| 5-15% | 1 (2.6) | 1 (6.3) | 0 (0.0) |
| 15-30% | 1 (2.6) | 0 (0.0) | 1 (4.5) |
| 30-50% | 3 (7.9) | 1 (6.3) | 2 (9.1) |
| >50% | 13 (34.2) | 9 (56.3) | 4 (18.2) |
| Testing not performed | 2 (5.3) | 0 (0.0) | 2 (9.1) |
| Don’t know | 9 (23.7) | 3 (18.8) | 6 (27.3) |
| **Institutional guideline on empiric antibiotic therapy for LOS** |  |  |  |
| Yes | 20 (52.6) | 4 (25.0) | 16 (72.7) |

NICU (neonatal intensive care unit); MRSA (methicillin-resistant *Staphylococcus aureus*); CoNS (coagulase-negative *Staphylococcus*); LOS (late-onset sepsis).
