# Supplementary material for: Empiric antibiotic prescribing practices for gram-positive coverage of late-onset sepsis in neonatal intensive care units in North America
Source: Infect Control Hosp Epidemiol. 2024 Nov 7;46(1):102–4. doi: 10.1017/ice.2024.176 (PMC11717478; doi:10.1017/ice.2024.176)
Supplement: Petel et al. supplementary material 2 — Petel et al. supplementary material [file S0899823X24001764sup002.docx]

Supplementary Table 2. Empiric antibiotic practices

| **Antibiotic practice** | **Total (N=38), N (%)** | **Canada (N=16), N (%)** | **USA (N=22), N (%)** |
| --- | --- | --- | --- |
| **Empiric vancomycin use for late-onset sepsis** |  |  |  |
| If known MRSA colonization | 22 (57.9) | 10 (62.5) | 12 (54.5) |
| If critically-unwell | 16 (42.1) | 10 (62.5) | 6 (27.3) |
| In all patients | 9 (23.7) | 2 (12.5) | 7 (31.8) |
| If central venous catheter (CVC) present | 8 (21.1) | 4 (25.0) | 4 (18.2) |
| If previous CoNS infection | 8 (21.1) | 4 (25.0) | 4 (18.2) |
| If recent CVC within 48 hours | 7 (18.4) | 5 (31.3) | 2 (9.1) |
| If multiple vascular access attempts within 48 hours | 1 (2.6) | 1 (6.3) | 0 (0.0) |
| Other | 4 (10.5) | 2 (12.5) | 2 (9.1) |
| **Empiric antibiotic use in critically-ill infants with a CVC** |  |  |  |
| Vancomycin | 28 (73.7) | 14 (87.5) | 14 (63.6) |
| Aminoglycosides | 19 (50.0) | 10 (62.5) | 9 (40.9) |
| Anti-staphylococcal penicillin agents | 11 (28.9) | 3 (18.8) | 8 (36.4) |
| Cefotaxime | 10 (26.3) | 8 (50.0) | 2 (9.1) |
| Ceftazidime | 7 (18.4) | 0 (0.0) | 7 (31.8) |
| Cefepime | 7 (18.4) | 0 (0.0) | 7 (31.8) |
| Piperacillin-tazobactam | 2 (5.3) | 1 (6.3) | 1 (4.5) |
| Meropenem | 2 (5.3) | 2 (12.5) | 0 (0.0) |
| Ampicillin | 1 (2.6) | 0 (0.0) | 1 (4.5) |
| Cefazolin | 1 (2.6) | 0 (0.0) | 1 (4.5) |
| Other | 3 (7.9) | 2 (12.5) | 1 (4.5) |
| **Empiric antibiotic use in critically-ill infants without a CVC** |  |  |  |
| Aminoglycosides | 22 (57.9) | 12 (75.0) | 10 (45.5) |
| Vancomycin | 18 (47.4) | 9 (56.3) | 9 (40.9) |
| Anti-staphylococcal penicillin agents | 13 (34.2) | 4 (25.0) | 9 (40.9) |
| Cefotaxime | 9 (23.7) | 6 (37.5) | 3 (13.6) |
| Ampicillin | 7 (18.4) | 3 (18.8) | 4 (18.2) |
| Cefepime | 7 (18.4) | 0 (0.0) | 7 (31.8) |
| Ceftazidime | 6 (15.8) | 0 (0.0) | 6 (27.3) |
| Piperacillin-tazobactam | 1 (2.6) | 0 (0.0) | 1 (4.5) |
| Meropenem | 1 (2.6) | 1 (6.3) | 0 (0.0) |
| Cefazolin | 0 (0.0) | 0 (0.0) | 0 (0.0) |
| Other | 2 (5.3) | 2 (12.5) | 0 (0.0) |
| **Empiric antibiotic use for rule-out sepsis in infants with a CVC** |  |  |  |
| Aminoglycosides | 26 (68.4) | 14 (87.5) | 12 (54.5) |
| Anti-staphylococcal penicillin agents | 20 (52.6) | 9 (56.3) | 11 (50.0) |
| Vancomycin | 16 (42.1) | 5 (31.3) | 11 (50.0) |
| Ceftazidime | 5 (13.2) | 0 (0.0) | 5 (22.7) |
| Cefepime | 5 (13.2) | 0 (0.0) | 5 (22.7) |
| Cefotaxime | 3 (7.9) | 1 (6.3) | 2 (9.1) |
| Ampicillin | 2 (5.3) | 1 (6.3) | 1 (4.5) |
| Cefazolin | 0 (0.0) | 0 (0.0) | 0 (0.0) |
| Piperacillin-tazobactam | 0 (0.0) | 0 (0.0) | 0 (0.0) |
| Meropenem | 0 (0.0) | 0 (0.0) | 0 (0.0) |
| Other | 1 (2.6) | 1 (6.3) | 0 (0.0) |
| **Empiric antibiotic use for rule-out sepsis in infants without a CVC** |  |  |  |
| Aminoglycosides | 28 (73.7) | 14 (87.5) | 14 (63.6) |
| Anti-staphylococcal penicillin agents | 20 (52.6) | 8 (50.0) | 12 (54.5) |
| Ampicillin | 12 (31.6) | 6 (37.5) | 6 (27.3) |
| Vancomycin | 6 (15.8) | 2 (12.5) | 4 (18.2) |
| Ceftazidime | 5 (13.2) | 0 (0.0) | 5 (22.7) |
| Cefotaxime | 4 (10.5) | 1 (6.3) | 3 (13.6) |
| Cefepime | 3 (7.9) | 0 (0.0) | 3 (13.6) |
| Cefazolin | 0 (0.0) | 0 (0.0) | 0 (0.0) |
| Piperacillin-tazobactam | 0 (0.0) | 0 (0.0) | 0 (0.0) |
| Meropenem | 0 (0.0) | 0 (0.0) | 0 (0.0) |
| Other | 1 (2.6) | 1 (6.3) | 0 (0.0) |

MRSA (methicillin-resistant *Staphylococcus aureus*); CoNS (coagulase-negative staphylococci); CVC (central venous catheter).
